# Supplementary material for: Association between 3-Year Repetitive Isolated Hematuria and eGFR Deterioration in an Apparently Healthy Population: A Retrospective Cohort Study
Source: Int J Environ Res Public Health. 2022 Sep 12;19(18):11466. doi: 10.3390/ijerph191811466 (PMC9517453; doi:10.3390/ijerph191811466)
Supplement: Supplementary file 1 [file ijerph-19-11466-s001.zip › PersistentHematuria_Figures_sup.pdf]

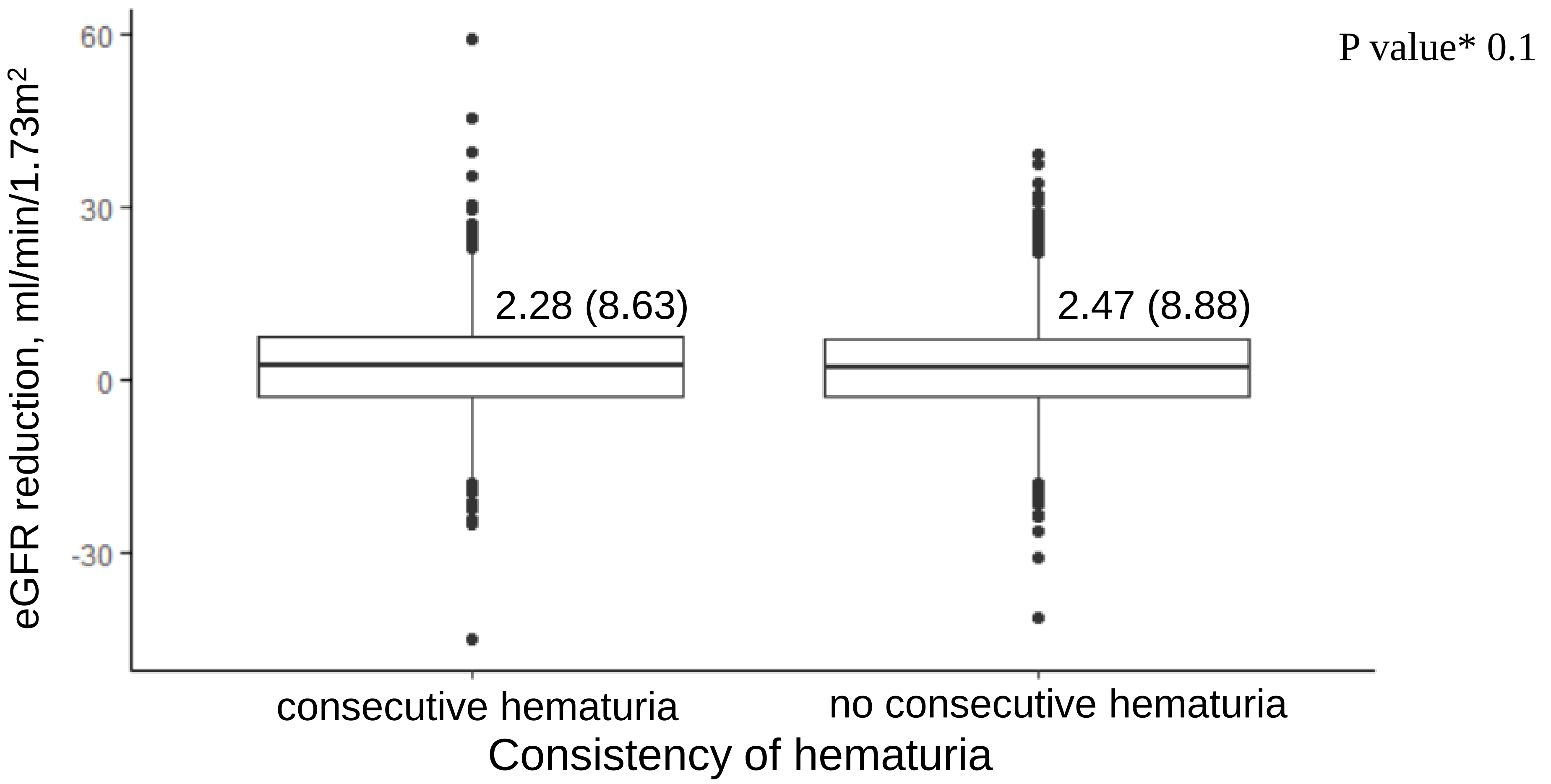

Figure S1: eGFR reduction at 5 years according to the consistency of hematuria

* Adjusted for age, sex, BMI, hypertension, diabetes mellitus, dyslipidemia, hyperuricemia, baseline GFR

(a) (b)

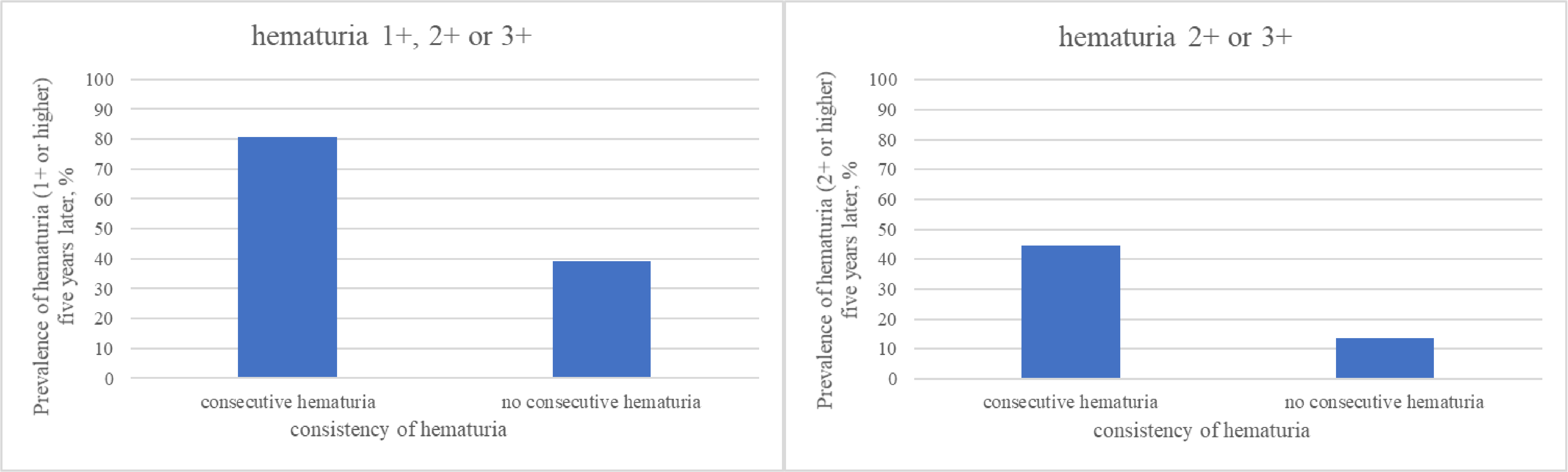

Figure S2: Prevalence of four urinary findings suggesting glomerulonephritis, in two groups that is stratified by the consistency of hematuria.
(a) hematuria 1+ or higher: 1+, 2+, or 3+, (b) hematuria 2+ or higher: 2+ or 3+,
(c) proteinuria, (d) hematuria and proteinuria

(c)

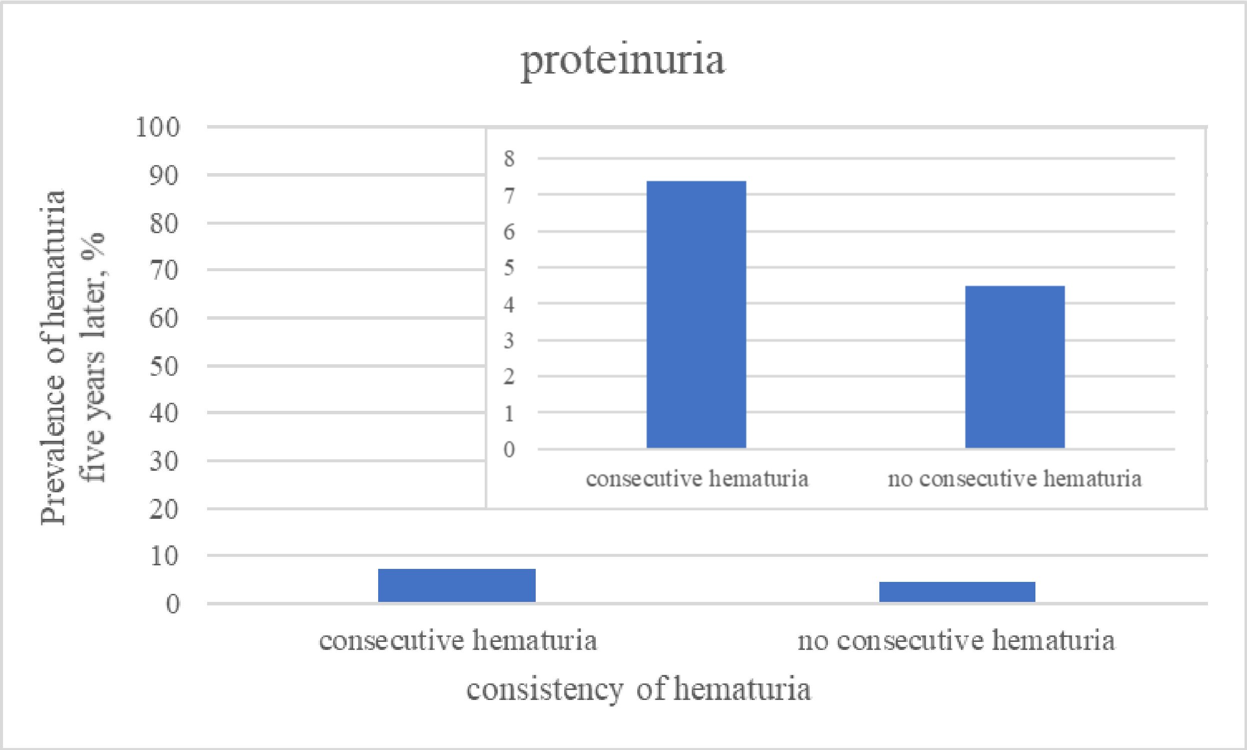

(d)

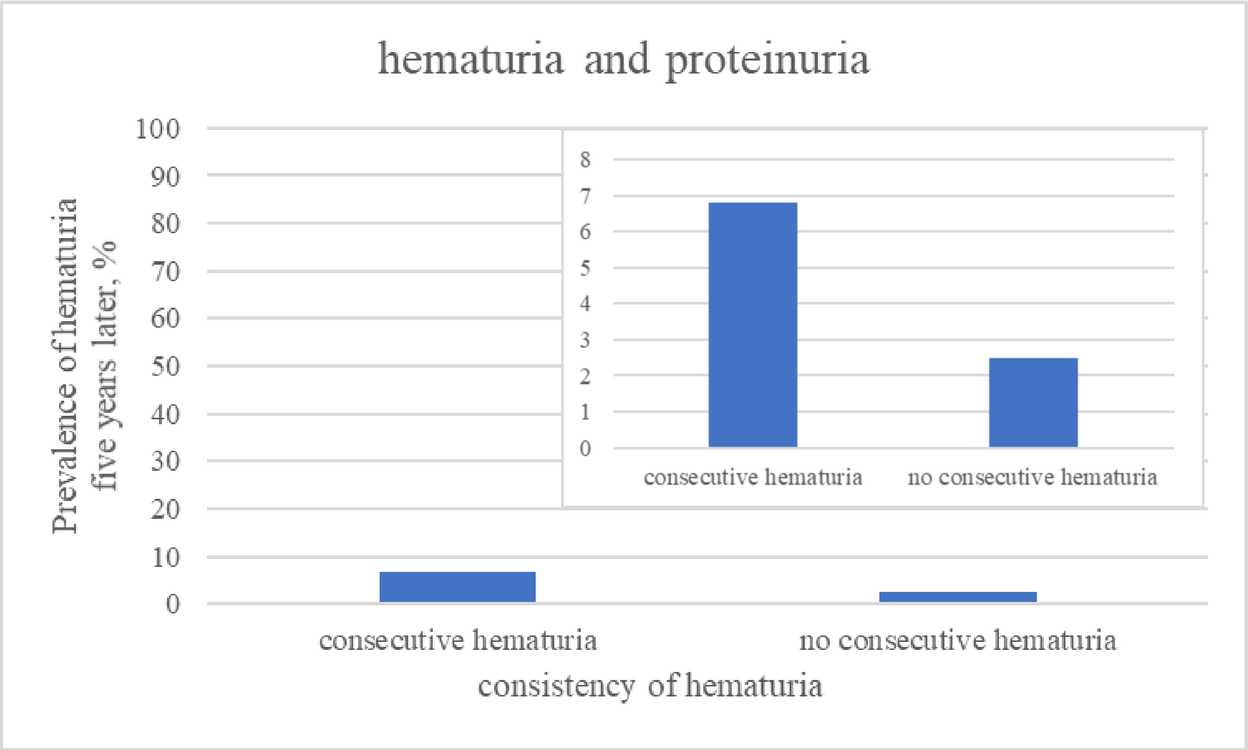

Figure S2: Prevalence of four urinary findings suggesting glomerulonephritis, in two groups that is stratified by the consistency of hematuria.
(a) hematuria 1+ or higher: 1+, 2+, or 3+, (b) hematuria 2+ or higher: 2+ or 3+,
(c) proteinuria, (d) hematuria and proteinuria
