# Supplementary material for: Association between 3-Year Repetitive Isolated Hematuria and eGFR Deterioration in an Apparently Healthy Population: A Retrospective Cohort Study
Source: Int J Environ Res Public Health. 2022 Sep 12;19(18):11466. doi: 10.3390/ijerph191811466 (PMC9517453; doi:10.3390/ijerph191811466)
Supplement: Supplementary file 1 [file ijerph-19-11466-s001.zip › PersistentHematuria_Tables_sup.pdf]

Table S1. (a) Association between urinary outcomes and the frequency of hematuria, stratified by age

	Prevalence					Risk ratio			
	thrice (n=680)	twice (n=429)	once (n=440)	never (n=555)	p value	thrice (n=680)	twice (n=429)	once (n=440)	never (n=555)
Hematuria, >=1+, n (%)									
age 20-39 y.o. (n=340)	59 (85.5)	52 (68.4)	32 (45.7)	28 (22.8)	<0.001	3.78 (2.70-5.32)	3.03 (2.11-4.34)	2.00 (1.32-3.02)	Ref
age 40-49 y.o. (n=795)	186 (81.2)	97 (63.8)	82 (42.1)	55 (25.6)	<0.001	3.16 (2.50-4.01)	2.50 (1.93-3.23)	1.62 (1.22-2.15)	Ref
age 50-65 y.o. (n=586)	322 (84.5)	143 (71.5)	77 (45.0)	44 (20.5)	<0.001	4.15 (3.18-5.42)	3.49 (2.65-4.61)	2.21 (1.62-3.02)	Ref
Hematuria, >=2+, n (%)									
age 20-39 y.o. (n=340)	34 (49.3)	23 (30.3)	11 (15.7)	10 (8.1)	<0.001	6.11 (3.22-11.60)	3.75 (1.90-7.45)	1.92 (0.86-4.30)	Ref
age 40-49 y.o. (n=795)	110 (48.0)	40 (26.3)	35 (17.9)	16 (7.4)	<0.001	6.43 (3.94-10.49)	3.54 (2.06-6.98)	2.38 (1.36-4.16)	Ref
age 50-65 y.o. (n=586)	180 (47.2)	59 (29.5)	19 (11.1)	11 (5.1)	<0.001	9.28 (5.17-16.66)	5.76 (3.12-10.65)	2.18 (1.07-4.46)	Ref

Table S1. (a) Association between urinary outcomes and the frequency of hematuria, stratified by age (continued)

	Prevalence					Risk ratio			
	thrice (n=680)	twice (n=429)	once (n=440)	never (n=555)	p value	thrice (n=680)	twice (n=429)	once (n=440)	never (n=555)
Proteinuria, >=1+, n (%)									
age 20-39 y.o. (n=340)	7 (10.1)	6 (7.9)	1 (1.4)	2 (1.6)	0.01	6.29 (1.34-29.45)	4.90 (1.01-23.64)	0.87 (0.08-9.46)	Ref
age 40-49 y.o. (n=795)	18 (7.9)	7 (4.6)	5 (2.6)	18 (8.4)	0.04	0.94 (0.50-1.75)	0.55 (0.24-1.28)	0.30 (0.11-0.80)	Ref
age 50-65 y.o. (n=586)	28 (7.3)	10 (5.0)	6 (3.5)	11 (5.1)	0.3	1.44 (0.73-2.84)	0.98 (0.42-2.25)	0.69 (0.26-1.83)	Ref
Hematuria and Proteinuria, n (%)									
age 20-39 y.o. (n=340)	6 (8.7)	4 (5.3)	1 (1.4)	2 (1.6)	0.06	5.39 (1.12-25.99)	3.26 (0.61-17.39)	0.87 (0.08-9.46)	Ref
age 40-49 y.o. (n=795)	17 (7.4)	5 (3.3)	3 (1.5)	10 (4.7)	0.03	1.59 (0.74-3.39)	0.71 (0.25-2.03)	0.33 (0.09-1.17)	Ref
age 50-65 y.o. (n=586)	26 (6.8)	6 (3.0)	4 (2.3)	5 (2.3)	0.02	2.95 (1.15-7.57)	1.29 (0.40-4.16)	1.01 (0.28-3.71)	Ref

Table S1. (b) Association between urinary outcomes and the frequency of hematuria, stratified by sex

	Prevalence					Risk ratio			
	thrice (n=680)	twice (n=429)	once (n=440)	never (n=555)	p value	thrice (n=680)	twice (n=429)	once (n=440)	never (n=555)
Hematuria, >=1+, n (%)									
male (n=1138)	267 (79.9)	135 (61.1)	95 (41.3)	72 (20.8)	<0.001	3.85 (3.11-4.77)	2.94 (2.33-3.70)	1.97 (1.52-2.55)	Ref
female (n=966)	300 (87.2)	157 (75.8)	96 (46.6)	55 (26.6)	<0.001	3.27 (2.60-4.12)	2.86 (2.25-3.63)	1.75 (1.33-2.29)	Ref
Hematuria, >=2+, n (%)									
male (n=1138)	141 (42.2)	56 (25.3)	34 (14.8)	20 (5.8)	<0.001	3.96 (2.85-5.50)	2.37 (1.62-3.47)	2.62 (1.82-3.79)	Ref
female (n=966)	183 (53.0)	66 (31.9)	31 (15.0)	17 (8.2)	<0.001	6.46 (4.05-10.29)	3.88 (2.36-6.38)	1.82 (1.04-3.19)	Ref
Proteinuria, >=1+, n (%)									
male (n=1138)	35 (10.5)	18 (8.1)	9 (3.9)	25 (7.2)	0.04	1.45 (0.89-2.38)	1.13 (0.63-2.02)	0.54 (0.26-1.13)	Ref
female (n=966)	18 (5.2)	5 (2.4)	3 (1.5)	6 (2.9)	0.08	1.80 (0.73-4.46)	0.83 (0.26-2.69)	0.50 (0.13-1.97)	Ref
Hematuria and Proteinuria, n (%)									
male (n=1138)	32 (9.6)	11 (5.0)	5 (2.2)	13 (3.7)	<0.001	2.56 (1.37-4.79)	1.33 (0.61-2.91)	0.57 (0.21-1.59)	Ref
female (n=966)	17 (4.9)	4 (1.9)	3 (1.5)	4 (1.9)	0.05	2.55 (0.87-7.48)	1.00 (0.25-3.95)	0.75 (0.17-3.31)	Ref

Table S2. Baseline characteristics according to the consistency of hematuria

	Total (n=2,104)	consecutive hematuria (n=853)	no consecutive hematuria (n=1,251)	P value
Age, years, mean (SD)	48.3 (8.22)	49.9 (8.06)	47.3 (8.17)	<0.001
Male, n (%)	1138 (54.1)	426 (49.9)	712 (56.9)	0.002
BMI, n (%)				0.04
<18.5	238 (11.3)	106 (12.4)	132 (10.6)	
>=18.5, <25	1,511 (71.8)	622 (73.0)	889 (71.1)	
>=25, <30	306 (14.6)	112 (13.1)	194 (15.5)	
>=30	48 (2.3)	12 (1.4)	36 (2.9)	
Dipstick hematuria, n (%)				<0.001
1+	1,348 (64.1)	433 (50.8)	915 (73.1)	
2+	639 (30.4)	364 (42.7)	275 (22.0)	
3+	117 (5.56)	56 (6.57)	61 (4.88)	
Serum creatinine, mg/dl, mean (SD)	0.74 (0.13)	0.734 (0.128)	0.740 (0.136)	0.4
eGFR, mL/min/1.73m^2, mean (SD)	80.0 (11.9)	78.55 (11.75)	81.03 (11.97)	<0.001
Hypertension, n (%)	651 (31.0)	283 (33.3)	368 (29.4)	0.07
Diabetes mellitus, n (%)	302 (14.4)	109 (12.8)	193 (15.5)	0.09
Dyslipidemia, n (%)	869 (41.4)	367 (43.1)	502 (40.2)	0.2
Hyperuricemia, n (%)	289 (13.8)	115 (13.5)	174 (13.9)	0.8

Abbreviations: BMI, body mass index; eGFR, estimated glomerular filtration rate

consecutive hematuria; 3 or more consecutive results, no consecutive hematuria; the others

Missing values: BMI: 1 (consecutive), hypertension: 4 (consecutive 3; no consecutive 1), diabetes mellitus: 2 (no consecutive), dyslipidemia: 3 (consecutive 1; no consecutive 2), hyperuricemia: 3 (consecutive 1; no consecutive 2)

Table S3. (a) Renal function, urinary findings and other outcomes at 5 years according to the frequency of hematuria

	thrice (n=680)	twice (n=429)	once (n=440)	never (n=555)	P value
Serum creatinine, mg/dl, mean (SD)	0.738 (0.150)	0.742 (0.150)	0.731 (0.141)	0.756 (0.154)	0.05
eGFR, mL/min/1.73m^2, mean (SD)	76.10 (12.26)	77.32 (12.89)	78.76 (12.22)	78.99 (13.58)	<0.001
eGFR, reduction mL/min/1.73m^2, mean (SD)	2.28 (8.66)	2.67 (9.31)	2.16 (8.14)	2.37 (8.83)	0.8
Dipstick hematuria, n (%)					
1+, 2+ or 3+	567 (83.5)	292 (68.2)	191 (43.8)	127 (23.0)	<0.001
2+ or 3+	324 (47.7)	122 (28.5)	65 (14.9)	37 (6.69)	<0.001
Dipstick proteinuria, n (%)	53 (7.81)	23 (5.37)	12 (2.75)	31 (5.60)	0.004
Hematuria and proteinuria, n (%)	49 (7.22)	15 (3.50)	8 (1.83)	17 (3.07)	<0.001
CKD, eGFR<60, n (%)	48 (7.06)	24 (5.56)	22 (5.00)	32 (5.77)	0.5
GFR 30% reduction, n (%)	4 (0.59)	5 (1.17)	3 (0.68)	3 (0.54)	0.7
Hypertension, n (%)	107 (15.7)	55 (12.8)	56 (12.7)	76 (13.7)	0.4
Glomerulonephritis, n (%)	6 (0.882)	6 (1.400)	1 (0.227)	1 (0.180)	0.07
ESKD, n (%)	0 (0)	0 (0)	0 (0)	0 (0)	
Urological diseases, n (%)	103 (15.1)	59 (13.75)	34 (7.73)	63 (11.4)	0.001

Abbreviations: eGFR; estimated glomerular filtration rate; CKD, chronic kidney disease; ESKD, end-stage kidney disease

Missing values: dipstick hematuria: 8 (thrice 1; twice 1; once 4; never 2), dipstick proteinuria: 6 (thrice 1; twice 1; once 3; never 1), hematuria and proteinuria: 8 (thrice 1; twice 1; once 4; never 2), hypertension: 4 (thrice 1; once 1; never 2)

Table S3. (b) Renal function, urinary findings and other outcomes at 5 years according to the consistency of hematuria

		consecutive (n=853)	no consecutive (n=1,251)	P value
Serum creatinine, mg/dl, mean (SD)		0.741 (0.147)	0.743 (0.154)	0.7
eGFR, mL/min/1.73m^2, mean (SD)		76.08 (12.13)	78.75 (13.12)	<0.001
eGFR reduction, mL/min/1.73m^2, mean (SD)		2.47 (8.88)	2.28 (8.63)	0.6
Dipstick hematuria, n (%)				
	1+, 2+ or 3+	687 (80.6)	490 (39.4)	<0.001
	2+ or 3+	380 (44.6)	168 (13.5)	<0.001
Dipstick proteinuria, n (%)		63 (7.39)	56 (4.49)	0.005
Hematuria and proteinuria, n (%)		58 (6.8)	31 (2.5)	<0.001
CKD, eGFR<60, n (%)		59 (6.92)	67 (5.36)	0.2
GFR 30% reduction, n (%)		7 (0.821)	8 (0.640)	0.6
Hypertension, n (%)		129 (15.1)	165 (13.2)	0.2
Glomerulonephritis, n (%)		8 (0.938)	6 (0.480)	0.3
ESKD, n (%)		0 (0)	0 (0)	
Urological Diseases, n (%)		129 (15.1)	130 (10.4)	0.001

Abbreviations: eGFR; estimated glomerular filtration rate; CKD, chronic kidney disease; ESKD, end-stage kidney disease

Missing values: dipstick hematuria: 8 (consecutive 1; no consecutive 7), dipstick proteinuria: 6 (consecutive 1; no consecutive 5), hematuria and proteinuria: 8 (consecutive 1; no consecutive 7), hypertension: 4 (consecutive 1; no consecutive 3)

Table S4. (a) Associations between outcomes and the consistency of hematuria, expressed by risk ratios

	Risk Ratio
Dipstick hematuria	
1+, 2+ or 3+	2.05 (1.90-2.21)
2+ or 3+	3.30 (2.82-3.87)
Dipstick proteinuria	1.65 (1.16-2.33)
Hematuria and proteinuria	2.73 (1.78-4.19)
CKD, eGFR<60	1.29 (0.92-1.81)
GFR 30% reduction	1.28 (0.47-3.53)

Abbreviations: eGFR; estimated glomerular filtration rate; CKD, chronic kidney disease; ESKD, end-stage kidney disease

Table S4. (b) Associations between outcomes and the consistency of hematuria, expressed by adjusted odds ratios

	Multivariate Odds Ratio*
Dipstick hematuria	
1+, 2+ or 3+	6.31(5.13-7.80)
2+ or 3+	5.17 (4.17-6.44)
Dipstick proteinuria	1.86 (1.27-2.74)
Hematuria and proteinuria	3.22 (2.05-5.13)
CKD, eGFR<60	0.90 (0.60-1.34)
GFR 30% reduction	1.41 (0.47-4.14)

Abbreviations: eGFR; estimated glomerular filtration rate; CKD, chronic kidney disease; ESKD, end-stage kidney disease

* Logistic regression analysis, adjusted for age, sex, BMI, hypertension, diabetes Mellitus, dyslipidemia, hyperuricemia, baseline GFR
